# Schema therapy versus cognitive behavioral therapy versus individual supportive therapy for depression in an inpatient and day clinic setting: study protocol of the OPTIMA-RCT

**DOI:** 10.1186/s12888-020-02880-x

**Published:** 2020-10-14

**Authors:** Johannes Kopf-Beck, Petra Zimmermann, Samy Egli, Martin Rein, Nils Kappelmann, Julia Fietz, Jeanette Tamm, Katharina Rek, Susanne Lucae, Anna-Katharine Brem, Philipp Sämann, Leonhard Schilbach, Martin E. Keck

**Affiliations:** 1 Max Planck Institute of Psychiatry, Kraepelinstraße 2-10, 80804 Munich, Germany; 2 International Max Planck Research School for Translational Psychiatry (IMPRS-TP), Munich, Germany; 3 University of Kassel, Kassel, Germany; 4 Berenson-Allen Center for Noninvasive Brain Stimulation, Division of Cognitive Neurology, Department of Neurology, Beth Israel Deaconess Medical Center, Harvard Medical School, Boston, MA USA; 5 University Hospital of Old Age Psychiatry, University of Bern, Bern, Switzerland; 6 Department of Neuropsychology, Lucerne Psychiatry, Lucerne, Switzerland; 7 Independent Max Planck Research Group for Social Neuroscience, München, Germany; 8 Ludwig-Maximilians-Universität, Munich, Germany; 9 Schmieder Hospital in Gailingen, Gailingen, Germany

**Keywords:** Depression, Randomized controlled trial, Psychotherapy, Schema therapy, Cognitive behavioral therapy, Supportive therapy, Treatment prediction, Relapse prevention, Mechanisms of change, Personalized psychiatry

## Abstract

**Background:**

Major depressive disorder represents (MDD) a major cause of disability and disease burden. Beside antidepressant medication, psychotherapy is a key approach of treatment. Schema therapy has been shown to be effective in the treatment of psychiatric disorders, especially personality disorders, in a variety of settings and patient groups. Nevertheless, there is no evidence on its effectiveness for MDD in an inpatient nor day clinic setting and little is known about the factors that drive treatment response in such a target group.

**Methods:**

In the current protocol, we outline OPTIMA (OPtimized Treatment Identification at the MAx Planck Institute): a single-center randomized controlled trial of schema therapy as a treatment approach for MDD in an inpatient and day clinic setting. Over the course of 7 weeks, we compare schema therapy with cognitive behavioral therapy and individual supportive therapy, conducted in individual and group sessions and with no restrictions regarding concurrent antidepressant medication, thus approximating real-life treatment conditions. *N* = 300 depressed patients are included. All study therapists undergo a specific training and supervision and therapy adherence is assessed. Primary outcome is depressive symptom severity as self-assessment (Beck Depression Inventory-II) and secondary outcomes are clinical ratings of MDD (Montgomery-Asberg Depression Rating Scale), recovery rates after 7 weeks according to the Munich-Composite International Diagnostic Interview, general psychopathology (Brief Symptom Inventory), global functioning (World Health Organization Disability Assessment Schedule), and clinical parameters such as dropout rates. Further parameters on a behavioral, cognitive, psychophysiological, and biological level are measured before, during and after treatment and in 2 follow-up assessments after 6 and 24 months after end of treatment.

**Discussion:**

To our knowledge, the OPTIMA-Trial is the first to investigate the effectiveness of schema therapy as a treatment approach of MDD, to investigate mechanisms of change, and explore predictors of treatment response in an inpatient and day clinic setting by using such a wide range of parameters. Insights from OPTIMA will allow more integrative approaches of psychotherapy of MDD. Especially, the identification of intervention-specific markers of treatment response can improve evidence-based clinical decision for individualizing treatment.

**Trial registration:**

Identifier on clinicaltrials.gov: NCT03287362; September, 12, 2017

## Background

In recent years, major depressive disorder (MDD) has become one of the three leading causes for years lived with disability with more than 264 million people affected worldwide [1]. For those affected, depression means personal suffering, reduced functioning and quality of life, social withdrawal, risk for co-morbid medical condition and increased mortality risk [2, 3]. High life time prevalence, ranging from 11.1 to 14.6% across countries [2], stresses the necessity for the development of effective forms of treatment.

The two main treatment approaches addressing MDD are antidepressant medication (ADM) [4] and psychotherapy [5, 6]. Even though, both approaches are effective, there is room for improvement, considering up to 50% of patients are non-responders to psychotherapy or ADM [7–9] and there is a high relapse rate of 54% within 2 years [10]. Alongside the development of innovative pharmacological approaches such as anti-inflammatory drugs [11, 12], the field of psychotherapy has also evolved considerably and introduced new forms of treatment for depression such as mindfulness-based cognitive therapy [13], the cognitive behavioral analysis system of psychotherapy [14] or schema therapy (ST) [15]. The latter has become increasingly popular within the last two decades and is the focus of the current study.

### The effectiveness of schema therapy

Besides its high rates of recurrence and non-responders to therapy, MDD is characterized by its heterogeneity of symptoms [16] and comorbidities on axis I and II [17–19]. Especially personality disorders are highly prevalent in inpatient settings [20], they increase the time for remission [21], and lower positive outcome in the treatment of MDD [22]. ST was originally conceptualized for non-responders of cognitive therapy and patients suffering from personality disorder (PD) [15]. Bamelis and colleagues found first indices for ST as treatment for personality disorders to reduce depressive symptoms as a secondary outcome at follow up [23]. Considering further evidence regarding ST for the treatment of MDD [24–26] we assume ST to be a promising approach in treating depression and overcoming weaknesses of CBT approaches. This applies especially for more severe and complex manifestations of MDD including particularly those with comorbid personality disorders as they represent clinical reality in inpatient and day clinic settings. The theoretical concept and practical clinical realization of ST represents an advancement of cognitive behavior therapy (CBT) and is based on psychological learning and attachment theories [15, 27]. Central to ST is the idea of early maladaptive schemas (EMS), which are defined as patterns of interpersonal learning experiences from (early) childhood, that are assumed to fundamentally shape human perception and psychological experiences and to play a key role in the development and maintenance of mental disorders [15]. Meanwhile, and different from earlier conceptualizations of schemas [28], EMS are understood as to comprise not only cognitive, but also emotional and physiological components. ST in turn focuses on the modification of such EMS and on the fulfillment of unmet emotional core needs. It uses key instruments including emotion-focused techniques, e.g., imagery rescripting and mode dialogues on chairs, and techniques to establish a certain form of therapeutic alliance between therapist and patients, so called “limited reparenting” [15].

Besides its increasing popularity in clinical practice, ST has also gained attention regarding its empirical foundations. First applied in the field of PD, ST has been proven to be very effective for borderline and cluster C personality disorders [23, 29–34]. Over the last two decades, the ST concept has been extended and transferred to different disorders such as already mentioned mood disorders [24–26], anxiety disorders [35, 36], posttraumatic stress disorder [37], eating disorders [38, 39] such as binge eating, obsessive compulsive disorder [40], disruptive behavior disorders [41], autism spectrum disorders [42], or rather process-oriented transdiagnostic features of psychopathologies such as emotional dysregulation [43, 44]. In this context, ST was administered in different settings, mostly to out-patients [45], partly to in-patients [46, 47], to specific age-groups such as older adults [48, 49], or forensic settings [50], and in different forms, such as individual therapy, group therapy [35, 46, 51, 52] or its combination [53]. Even though there is a growing body of literature and evidence for the effectiveness of ST, only few of the trials and studies have provided reliable and transferable results. This is mostly due to 1) small sample-sizes [25, 26, 51], 2) inadequate trial designs because of missing randomization and/or control conditions [40, 42, 54], 3) inadequate control groups [37], or 4) missing blindness of raters [29, 30, 41].

We aim to avoid these shortcomings using a large-scale RCT (target *N* = 300) to test the overall effectiveness of ST as a treatment of MDD in an inpatient and day clinic setting. Participants are randomized to either ST, CBT as an established form of psychotherapy or individual supportive therapy (IST) as a nonspecific, active treatment that serves as a control arm for nonspecific factors of psychotherapy.

### Mechanisms of change in schema therapy

Within the last decades, psychotherapy research proceeded on from pure outcome research to process oriented research in order to identify the mechanisms of change (MOCs), which are assumed as the active ingredients in psychotherapy. It is debated which factors might actually make psychotherapy work – mechanisms that all therapies have in common or mechanism that differentiate between various approaches [55–57].

MOCs are the theory-driven reasons for change in therapy, such as specific events or processes, from a methodological perspective considered as mediators of the effect of a treatment (e.g. ST) on an outcome (e.g. symptom severity) [58, 59]. When it comes to the research of mediators of psychotherapy for MDD, so far, mostly cognitive factors, such as dysfunctional attitudes, automatic thoughts, or rumination have been examined in mostly cognitive-behavioral treatment approaches [60–62]. Less is known about MOCs in supportive treatments like IST [63, 64], and except from single studies [65, 66], hardly anything is known about the driving factors of schema therapy.

Beyond testing the effectiveness of ST, we therefore aim in the current trial to examine the underlying MOCs of ST and to investigate if and how they differ from those operating in CBT and IST. Following recommendations in the literature [57, 62], we include multiple alternative potential mediators of therapeutic change in a fine grained temporal design. In order to detect their temporal relationships, we use repeated and continuous measures of outcome and treatment specific factors which we assume being particularly relevant in one of the two active treatment arms: e.g. schema related factors like EMS (ST), cognitive factors like dysfunctional attitudes (CBT), or non-specific factors like goal setting in a common factor treatment condition (IST) (for details see below).

### Exploring predictors of treatment response

The development and testing of new psychotherapy approaches and the identification of relevant processes and MOCs are two useful strategies in order to approach the mentioned problem of high rates of non-responders and relapse regarding the treatment of MDD. Another possibility to increase positive treatment outcome is to get beyond average effectiveness in order to better understand “what works for whom?” [67]. The call for personalized medicine for depression [68] that tailors down therapy to the individual needs of patients, has generated different approaches to identify predictors of treatment repsonse.

These approaches investigate *general* (or prognostic) predictors of outcomes independent from treatment type as well as *differential* (or prescriptive) moderators that discriminate responses between treatment types [68, 69]. This refers for example to ADM versus psychotherapy [70–72], different types of ADM [73, 74], or different types of psychotherapy [75, 76].

In the past, parameters from different domains such as genetics and epigenetics [77], inflammation [12], neuroimaging [78], self-reports including clinical parameters, or sociodemographic variables [69, 79, 80] have been used to identify predictors of treatment response or outcome. Due to the heterogeneity and complexity of MDD, single domain approaches just implementing one kind of parameter were of limited success [81]. Therefore, in the recent years, the field of personalized medicine and precision psychiatry proceeded to the application of artifical intelligence and machine-learning models [82, 83] combining multi-variate data sets [75], and implementing biological and clinical patient profiles [84] in order to predict treatment outcome for psychiatric disorders in general, and MDD in particular.

In the current research, we follow this strategy and apply a similar multi-variate approach as it was suggested in the past [83, 85, 86] by including variables from self-reports (e.g. symptoms or personality traits), sociodemographics (e.g. age, gender), clinical data (e.g. comorbidities), neuropsychology (e.g. cognitive impairment), electrocardiography (ECG), biological parameters (e.g. genetics), and neuroimaging (for details see below). By doing so, we aim to explore general predictors of treatment outcome in a combined pharmaco-psychotherapy-setting as well as examining differential predictors of specific relevance for ST.

## Objectives

Taken together, the OPTIMA-Trial addresses three major objectives: *First and main aim is* to investigate the immediate and long-term effectiveness of ST compared to IST and CBT for moderate to severe MDD in an inpatient and day clinic setting using among others depression self-reports, clinical assessments, and recovery rates (for details regarding measures and hypotheses see section below). *Second*, the OPTIMA-Trial examines the role of specific MOCs which we assume to be particularly relevant for the two active treatments arms (ST and CBT), and non-specific MOCs, which we expect to play a role in psychotherapy in general independently from specific intervention techniques. *Third*, the OPTIMA-Trial aims to identify predictors of treatment response among a variety of clinical, (neuro-) psychological, biological, physiological, and neuroimaging parameters. Due to the specific characteristics of the current setting and design (see below), we do not have definite preformulated hypotheses for the interplay of these variables and follow an exploratory approach.

## Methods

### Study design

The OPTIMA study is a clinical RCT, in which participants are randomized in a parallel group design to one of the three treatment arms ST vs. CBT vs. IST using computer generated numbers in a block randomization technique with 1:1:1 allocation. Allocation is concealed for raters at any time, for participants and therapists before the day of the first psychotherapeutic session. The trial is registered at clinicaltrials.gov (NCT03287362). The study protocol was approved by the Institutional Ethic Committee of the Faculty of Medicine of the Ludwig-Maximilians-University Munich (Project number 17–395). All participants provided written informed consent prior to clinical interviews, further measures and randomization.

### Setting, recruitment and enrollment

The study is conducted at the hospital of the Max Planck Institute of Psychiatry in Munich, Germany, in an inpatient and day clinic setting. Participants are recruited from the overall patient population typically treated at the hospital, which covers a wide range of diagnoses with a focus on stress-related disorders such as MDD and anxiety disorders. Thus, we aim to enroll a heterogeneous sample that corresponds to clinical reality. Furthermore, we recruit patients through flyers and information events from collaborating institutions and outpatient practices.

During an obligatory clinical admission interview, all patients that are assigned to the involved wards and day clinics are screened for inclusion and exclusion criteria by a physician who also decides on the main diagnosis. When patients meet all criteria and report a general willingness to participate, they are informed in detail by a specially trained study assistant (psychologist). The study assistant explains in oral and written form about study procedures, such as randomization, timeline of measures, treatment forms and duration of the study and the patient is given the possibility to ask questions and consider participation.

After providing written informed consent, baseline assessment is completed in the first study week, participants are assigned to one of the three treatment arms (ST vs. CBT vs. IST) using block randomization with a 1:1:1 allocation ratio stratified by treatment units (wards and day clinics). Following the baseline assessments, the actual treatment phase lasts for 7 weeks. After a naturalistic phase of 6 months and 2 years following study completion, participants are re-invited to visit the hospital for follow-up assessments, a psychotherapy individual session and/or filling in online-questionnaires.

**Fig. 1:**
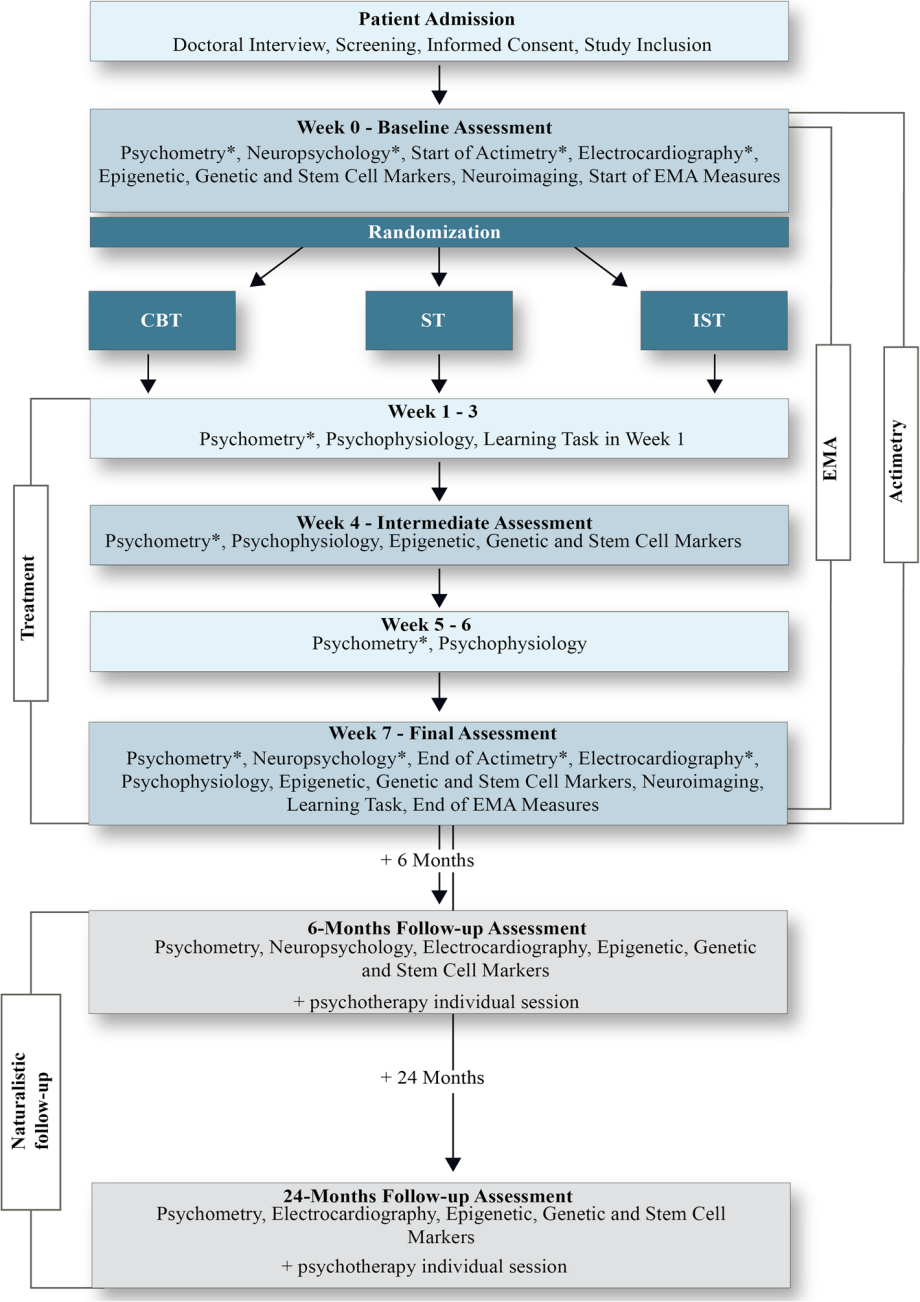
Flow chart of enrollment and assessments of the OPTIMA-Study. Note: *Assessment domains are obligatory for participation. EMA – Ecological Momentary Assessment

The participation in the study is fully voluntary. Withdrawal from the study is possible at any time and does not affect access to treatment. The OPTIMA-Trial started in September 2017 and is expected to be finished (including follow-up measurements) by the end of 2022. For details of the patient enrollment, assessments and intervention see Fig. 1.

### Inclusion and exclusion criteria

Inclusion criteria are 1) main diagnosis of MDD, single episode or recurrent, moderate or severe (Beck Depression Inventory-II (BDI-II) - score ≥ 20 [87] or Montgomery-Asberg Depression Ratings Scale (MADRS)-score ≥ 20 [88]), without psychotic symptoms representing ICD-10 diagnoses (F32.1, F32.2, F33.1, or F33.2, 2) age between 18 and 75 years, and 3) informed consent to the study procedures and assessments in written form.

Exclusion criteria are 1) diagnosis of MDD, single episode or recurrent, severe with psychotic symptoms (F32.3, F33.3 according to ICD-10), 2) acute suicidality, 3) lifetime history of any psychotic or bipolar disorder, 4) severe neurological or internal concomitant or past diseases, 5) an IQ < 80 and/or severe learning disability, 6) current alcohol or any illicit drug withdrawal syndrome, 7) concomitant organic mental disorder (F00 – F09 according to ICD 10), 8) concomitant substance induced disorders, 9) severe mutism or stupor, 10) mental disorder secondary to a medical condition or substance use disorders, 11) pregnancy and lactation period, and 12) missing eligibility for psychotherapy due to language barriers.

Drop-outs during the conduct of the study were defined as enrolled participants 1) who withdraw informed consent, 2) for whom exclusion criteria became known to be fulfilled during the conduct of the study (such as a bipolar-diagnosis in the previous medical history discovered during the trial), or 3) who missed more than six sessions of psychotherapy (corresponds to 22% of the overall psychotherapy dose). To sum up, we anticipate these criteria to result in a heterogeneous sample that corresponds to the clinical reality of a psychiatric hospital.

### Interventions

#### Schema therapy

ST represents a transdiagnostic psychological treatment rooted in cognitive therapy that integrates elements of several different psychotherapeutic approaches including gestalt therapy, psychodynamic therapy, and ego-state therapy [15] . Key aspects that distinguish ST from traditional CBT are a) a central and primary development-based focus on emotional needs and on the role of EMS in the development and maintenance of psychopathology, b) the additional application of experiential and emotion-focused techniques, c) the use of schema modes, which refer to moment-to-moment emotional, cognitive, physiological states and coping responses, and d), a strong emphasis on the importance of the therapeutic relationship as a means for therapeutic change with therapists taking a stance that has been described as “limited re-parenting” [15].

The ST treatment manual in the OPTIMA-Trial [89] includes three phases as a combined group- and individual session concept: In a first exploration phase patients are introduced to the main concepts of ST. Their predominant schemas and modes are explored and identified. Therapy goals are set and the therapeutic alliance is established. The second phase focuses on the change of EMS and dysfunctional and maladaptive modes by applying specific interventions such as mode dialogues on chairs, imagery rescripting, validation and empathic confrontation, and limited reparenting. The ST-manual did not include cognitive restructuring in a CBT sense. The third phase addresses strategies for transfer and relapse prevention.

#### Cognitive behavioral therapy

CBT combines aspects of behavioral therapy [90] and cognitive therapy [91] and was originally designed for the treatment of MDD based on Beck’s theory of depression [28, 92]. CBT is recommended as first-line treatment for individuals with mild to moderate MDD (National Institute for Clinical Excellence NICE, 2004) and recommended for severe and chronic depression in combination with psychopharmacological treatment [93].

The CBT sessions in the OPTIMA-Trial are conducted according to a treatment manual that is conceptualized as a combined group-and individual session cycle and represents a modified and extended version of a different well-established and widely used treatment concept [94]. The manual includes five main modules for the group-concept dealing with the following topics: i) psycho-education on MDD and the treatment concept, ii) behavioral activation, iii) the modification of dysfunctional attitudes and automatic thoughts, iv) social competence training, and v) relapse prevention. These group modules are transferred to the single sessions.

Considering ST as a further development from C(B) T, both approaches share some common roots [15], even though the practical implementations differ. In both cases, dysfunctional core beliefs respectively schemas play a central role in the emergence and maintenance of MDD. In the used manuals, they are targeted by different means such as cognitive restructuring (CBT) and mode dialogues on chairs or imagery work (ST). Both approaches share the idea of making a psychoeducational model transparent in the beginning of every treatment circle. Additionally, both use behavioral experiments in order to establish adaptive and overcome maladaptive behavior patterns. In the used manuals, these interventions are part of the behavioral activation and social competence training (in CBT) and the transfer phase (in ST).

#### Individual supportive therapy

IST can be considered as an active and nonspecific, non-ST and non-CBT approach. Following the concept of a bio-psycho-social disease model of MDD, IST is based on the common factors of psychotherapy, which include among others support of the patient, therapeutic alliance, activation of resources, actualization of the patient’s problems, motivational clarification, and problem solving [95, 96]. These factors can be fostered from the therapist side through techniques like being supportive, applying an understanding and acknowledging conversational style, calming assertions, putting the focus on perceiving progress, or using interested inquiries. The IST manual is based on three pillars: the patients’ personal resources, the therapeutic alliance between him/her and the therapist, and the focus on emotions. Regarding the latter, patients are encouraged and supported to give space to emotions, whenever they are experienced during the psychotherapy session. This happens without IST being based on a psychodynamic frame work such affect phobia treatment [97] or emotions being the foundational and central subject matter of the treatment like in emotion-focused therapy [98].

Therapists are instructed to apply a supportive, non-judgmental, open, empathic, tolerating, positive communication style. The IST-program uses the insights of client-centered communication skills. Patients define the topics and content of each session by themselves. The therapists are instructed not to use specific psychotherapeutic intervention strategies from ST or CBT. Similar concepts of supportive therapy have been used elsewhere [99] as an active control arm that takes into account these important carriers of interpersonal medicine in general and have been shown to be effective in the treatment of MDD.

In order to avoid and control for different expectancy effects between treatment arms, therapists in the IST condition are encouraged to introduce participants into the concept and common factors of IST. Additionally, therapy expectations are assessed using Patient Questionnaire on Therapy Expectation and Evaluation [100] and considered in statistical analysis.

#### Psychotherapy dose and duration

The psychotherapy in all three arms (ST, CBT, IST) is offered in accordance with a guideline-adherent psychiatric-psychotherapeutic hospital care for depression [101] as a combined concept including two group- (100 min. each) and two individual sessions (50 min. each) per week applied over the course of 7 weeks. The group-sessions are offered in a “semi-open” manner in order to keep the maximal group size stable (eight participants). That is, newly randomized participants are integrated in the ongoing group-session circle by the beginning of a treatment week. The received amount of treatment sessions of each participant is documented, so it can function as a control variable in the statistical analysis, since it might differ from the protocol guidelines for various reasons. Six and 24 months after study completion, participants are invited to a follow-up measurement appointment and are offered an additional individual psychotherapy session after the measurements have been taken.

#### Therapist training and monitoring of adherence

Psychotherapy is conducted by clinical psychologists and psychiatrists who receive a comprehensive in-house training including multiple workshops by leading experts to ensure high qualification in their performance in a specific treatment arm. Study therapists are supervised by external experts on a monthly schedule. Additionally, all study therapists meet every 3 months to ensure reliable administration of techniques across units. In order to assure the integrity of the specific psychotherapy, all sessions are videotaped and a random selection of tapes rated for adherence to the manuals and its specifications. Based on existing adherence scales [99, 102] (Zwick J, Hautzinger M: Manual zu Einschätzung der Manualadhärenz und Kompetenz der Psychotherapeuten im Rahmen der A2-Bipolife-Interventionen, unpublished), we developed a 13-item adherence-scale (four items on ST, five items on CBT, four items on IST) with each item rated on a six-point Likert-scale. Two independent raters are trained and interclass correlation coefficients are being calculated to ensure sufficient inter rater reliability.

Study-therapists are allocated to one specific active psychotherapy arm (ST or CBT) and the nonspecific IST-arm. In order to avoid the confusion of the specific psychotherapy arms (ST and CBT), the assignment of a therapist is assured for at least 1 year. All therapists are encouraged to adhere to the particular manual.

### Blinding

Clinical interviews and ratings are conducted by raters, trained in workshops and individual sessions, evaluated and supervised, who are blind to the psychotherapy arm [103]. In case of an unblinding of a rater, corresponding ratings are conducted by another member of the rating team, thus assuring blinding throughout the sample. Interrater reliability between raters is assessed intermittently based on the ratings of the same patient by all raters. After evaluation, an additional training is offered as needed.

### Concomitant care

In order to protect against biases, all study participants are asked not to join any other psychotherapeutic program such as mindfulness training. Potential influencing factors that form part of an inpatient/day clinic treatment program such as Sports therapy, case management, or ergotherapy are documented for later use in the statistical analysis as potential confounder.

The OPTIMA study design does not regulate parallel psychopharmacotherapy but leaves the decisions hereon to the psychiatrist in charge. The psychopharmacological substances a patient receives throughout the intervention phase, however, is documented in terms of type and dosage.

## Measures and hypotheses

### Primary and secondary measures for outcome comparison

We choose BDI-II [87] as primary outcome to capture the change of symptom severity over the course of 7 weeks. BDI-II represents a widely used and well established self-assessment of MDD [104, 105] and thus assures comparability of results with former research. In order to overcome some of BDI-II related restrictions such as sensitivity to maladaptive personality traits [106], we added MADRS [88], as clinical assessment of change in MDD as secondary outcome. Further secondary outcomes are recovery rate (change in diagnosis) measured by the Munich-Composite International Diagnostic Interview (M-CIDI) [107], change in general psychopathology using the Brief Symptom Inventory (BSI) [108], change in global functioning and quality of life according to the World Health Organization Disability Assessment Schedule (WHODAS) [109] and the World Health Organization Quality Of Life (WHO-QOL) assessment [110]. Further secondary outcome measures are drop-out rates, and remission rates. Based on previous research, our main hypotheses are that ST including its intervention techniques is more effective (superior) in the treatment of MDD compared to IST as a nonspecific-common factor psychotherapy [111] (H1), and 2) ST is non-inferior compared with CBT regarding treatment response and recovery rates operationalized by primary and secondary outcomes after the intervention and after 6 months [24] (H2).

### Further measures of potential MOCs for process comparison

In order to delineate the MOCs of ST and compare them with underlying mechanisms of CBT and IST, we include multiple potential mediators, most of them measured in weekly intervals (cf. [62]). Depending on treatment condition, we hypothesize different MOCs to be relevant.

In accordance with the theoretical concept of ST, we consider schemas (Young Schema Questionnaire – Short Version 3) [112], Young Positive Schema Questionnaire) [113], modes (Schema Mode Inventory) [114], therapeutic alliance (Working Alliance Inventory) [65, 112], attachment style (Relationship Scales Questionnaire) [115], affect (Need for Affect Questionnaire [116], the Positive and Negative Affect Schedule) [117, 118], and emotion regulation (Emotion Regulation Questionnaire) [119, 120] to function as MOCs of ST regarding the change in MDD (H3).

Based on previous research [62] and in contrast to ST, for CBT we assume cognition related aspects of depression (Automatic Thought Questionnaire [121], Dysfunctional Attitude Scale [122], Cognitive Style Questionnaire – Short Form in German [123], Internal and External Control Beliefs Scale [124], and self-efficacy measured by *Allgemeine Selbstwirksamkeitsskala* [125]) to mediate change in MDD (H4).

Additionally, we expect all three conditions, but especially the non-specific treatment arm (IST) to work through common factors of psychotherapy, such as goal setting (Goal Attainment Scale) [126], therapy expectations (Patient Questionnaire on Therapy Expectation and Evaluation) [100], further general mechanisms (Scale for the Multiperspective Assessment of General Change Mechanisms in Psychotherapy) [127], and non-specific session characteristics (Session Evaluation Questionnaire) [128] to be associated with change (H5).

In order to examine temporal and causal relations between MOCs and outcomes measures, measurements should happen at the same time point [55, 56]. We therefore include outcome measures (BDI-II, BSI) and MDD related constructs such as thought-action fusion in the context of suicidality [129], resilience (Brief Resilience Scale) [130], perceived stress (Perceived Stress Scale) [131, 132], and coping with depressive symptoms (Response Style Questionnaire) [133] on a weekly base. See Additional file 1: Appendix A for details regarding time points of measurement.

Beyond that, the exploration of potential MOCs of ST should not be restricted to weekly self-reports, but also include information from other sources in order to get a more comprehensive understanding of the course and processes during psychiatric care [62]. Therefore, we complement the set of weekly self-report variables by adding actimetry measures to assess locomotor activity, psychophysiological measures during the actual psychotherapy session, and continuous measures of depression-related parameters such as mood and repetitive negative thinking:

#### Actimetry

Changes in locomotor activity and unbalanced rest-activity cycles are widely known as key features of depression [134, 135]. Actimetry provides an objective and unobtrusive mean of assessing sleep and activity with high temporal resolution, so that activity- and sleep-related symptoms (e.g., insomnia) and potential treatment effects (e.g. change in activity) can be dynamically captured [136].

In the current study, locomotor activity and sleep behavior are assessed using actimetry wrist-watch devices. The devices are worn by participants throughout the treatment phase of the study over the course of 7 weeks continuously except during activities that may damage the device or be a risk to participants (e.g., swimming/contact sports).

#### Psychophysiology

Psychophysiology in general and interpersonal physiology in particular, is related to psychotherapy processes [137, 138] which are specifically relevant in ST [15], such as therapeutic alliance [139] and emotion regulation techniques applied by the therapist [140]. We therefore assess physiological parameters such as heart-rate (HR), electrodermal activity, and body temperature in a sub-sample of patients during psychotherapy sessions through hand-wrist devices that are worn by patients and therapists. Thus, we aim to gain insight into the synchronicity of physiological processes underlying ST specific intervention techniques, e.g. imagery rescripting, and to investigate how these processes differ from CBT techniques such as cognitive modification [141].

#### EMA of depressive core symptoms and repetitive negative thinking (RNT)

During the seven-week treatment phase, patients are asked to participate in an app-based EMA [142] that acquires momentary states of different core symptoms of MDD (e.g., mood, withdrawal) and RNT [143]. EMA offers several advantages compared to traditional questionnaire assessments, which are particularly relevant for the investigation of clinical processes, such as being closer in time to the experienced phenomenon, reducing recall bias (specifically relevant for MDD samples) [144], collecting data in naturalistic settings [145], and examine within-person processes which are especially important in psychotherapy. The measurements take place three times a day and comprise a total of eleven items. The app is installed on the patient’s personal smartphone or a provided device.

Such high-frequency measures of depressive symptoms and RNT can provide new insights into the dynamic changes over the course of the treatment phase and are particularly useful, given the variability of symptoms within and between days and individuals.

### Further measures for exploring treatment prediction

Beside outcome and process comparison, the current study includes a variety of potentially MDD-related predictors from the domains of neuropsychology, ECG, biology, and cognitive and social neurosciences in order examine patterns of patient characteristics using an exploratory approach. For an overview of all assessment domains and time points of measurements, see Table 1. Some of the listed measures are obligatory, while others are optional sub-studies that are not applied to all participants (see Table 1). In order to ensure the practicability and implementation of the measures in clinical routine, a feasibility study was conducted prior to the start of the actual trial and subsequently, processes were adapted if necessary.

**Table 1:** Overview on time points of measurement

Study week	T0	T1	T2	T3	T4	T5	T6	T7	T8	T9
		Treatment phase								
**Primary outcome**										
BDI	x	x	x	x	x	x	x	x	x	x
**Secondary outcomes**										
MADRS	x				x			x	x	x
M-CIDI	x							x	x	x
WHODAS	x				x			x		
WHOQOL	x				x			x		
Self-reports	x	x	x	x	x	x	x	x	x	x
**MOCs and process measures**										
Self-reports	x	x	x	x	x	x	x	x	x	x
Actimetry^a^	xxx	xxx	xxx	xxx	xxx	xxx	xxx	xxx		
Psychophysiology		xx	xx	xx	xx	xx	xx	xx		
EMA	xxx	xxx	xxx	xxx	xxx	xxx	xxx	xxx		
**Potential predictors**										
Neuropsychology^a^	x							x	x	
ECG^a^	x							x		
Biological parameters	55ml				28ml			28ml	28ml	28ml
Neuroimaging	x							x		
Learning Task		x						x		

*Note:* T0 = baseline measures before treatment start; T1 = first study week; etc.; T8 = six months follow-up; T9 = 24 months follow-up; x = one time point of measurement per week; xx = two time points of measurement per week; xxx = quasi continuous data collection ranging from three times per day (EMA) to every 30 seconds (actimetry). ^a^Assessment domains are obligatory; Secondary outcome self-reports include BSI, WHODAS, and WHO-QOL; For further details on the MOC measures (particularly common factor measures) see Additional file 1: Appendix A.

*Note:* T0 = baseline measures before treatment start; T1 = first study week; etc.; T8 = 6 months follow-up; T9 = 24 months follow-up; x = one time point of measurement per week; xx = two time points of measurement per week; xxx = quasi continuous data collection ranging from three times per day (EMA) to every 30 s (actimetry). *Assessment domains are obligatory; Secondary outcome self-reports include BSI, WHODAS, and WHO-QOL; For further details on the MOC measures (particularly common factor measures) see Additional file 1: Appendix A.

#### Neuropsychology

Cognitive impairment plays a key role as a transdiagnostic factor in psychiatric disorders in general and MDD in particular [146–148]. These deficits affect different cognitive domains such as memory, executive functions, attention, and learning [149, 150] and in many cases outlast the remission of depressive symptoms [151, 152]. They function as a mediator of functional impairment in MDD in general [153] and thus, as potential predictor and working mechanism of treatment. Therefore, we assess cognitive functions in order to identify its influence on the outcome effects of treatment before treatment (T0), after treatment (T7), and 6 months after completion of the study at follow up assessment (T8). We assess three basic domains of cognitive processing: attention, executive functions, and memory. The cognitive test battery includes tests from the Test of Attentional Performance [154], which is used to assess cognitive inhibition (Go/No-go-Task), working memory (Dual n-back), and cognitive flexibility. The test battery “Materialien und Normwerte für die neuropsychologische Diagnsotik” [155] is administered to test episodic memory, word fluency and sensitivity to interference (Stroop task). Additionally, we collect information on attention and cognitive flexibility with the Trail Making Test (TMT) and the d2-R [156], and assess intelligence with the *“Mehrfachwahl-Wortschatztest”* [157].

#### Electrocardiography

Heart-rate variability (HRV) parameters carry important information on the status of the autonomous nervous system that is unstable in stress-related disorders [158, 159] and has been found to correlate with the severity of depressive symptoms [160]. Therefore, we included Two-channel mobile ECG is routinely obtained during standardized conditions (5 min of resting state, 1 minute of deep breathing, overnight measurement of twelfe hours) at T0 and T7 to extract of HR and HRV parameters.

#### Biological parameters

Biological parameters would be most welcome and important tools in predicting response to specific psychotherapeutic or psychopharmacological interventions [161]. Recently, genome-wide association studies for unipolar depression have revealed a number of significantly associated loci [162, 163] and epigenetic modifications are considered to play an important role in the pathogenesis and therapy response in patients suffering from this disorder [164]. Gene-environment-interactions such as the role of trauma exposure have been discussed to shed a light to the genetics of MDD [165]. Even though, there is an emerging body of research extending these gene-environment-interactions to learning-contexts of psychotherapy [166], so far there are no satisfying validated biological parameters to assist in the decision-making process regarding the best treatment option for patients suffering from MDD. Studies aiming to integrate underlying pathomechanisms in this process have been designed [167]. In this study, we are aiming to identify biological parameters to contribute to the understanding of the response to psychotherapy.

We test for biological parameters of therapy response according to current and future evidence from clinical and preclinical data. Serum and plasma samples are stored for the analyses of parameters possibly associated with therapy response. The possible levels of investigation include genetics, epigenetic measures such as deoxyribonucleic acid (DNA) methylation, non-coding ribonucleic acid (RNA) but also other epigenetic markers such as histone modifications as well as proteomics, gene expression and metabolomics. Blood is drawn before treatment start (T0), in treatment week 4 (T4) and after treatment (T7) as well as during follow-up assessments at 6 months and 24 months after end of treatment. The sample includes ethylenediamine tetra acetic acid (EDTA) blood for DNA extraction (genome-wide genotyping and DNA methylation), RNA tubes for microRNA expression as well as small non-coding RNAs and serum and plasma for proteomics and metabolomics. Plasma can also be used to assess miRNAs circulating in exosomes. Finally, only at the baseline visit, we collect peripheral blood mononuclear cells (PBMCs) using Ficoll separation. At least 30 Mio. cells are stored for each individual and these can be used as a source tissue for induced pluripotent stem cell programming as well as functional assays in live mononuclear cells. Cells are stored with dimethyl sulfoxide (DMSO) as the stabilizer in liquid nitrogen. Induced pluripotent stem cells are established from blood cells and tested. In addition, the patients receive the standard safety routine blood draws of the clinical routine. The total blood volume drawn before treatment (T0) is 55 ml, the total blood volume drawn after 4 weeks (time point T4), 7 weeks (T7) after 6 and 24 months (T8) is 28 ml. All samples are entered into the biobank at the Max Planck Institute of Psychiatry which has been approved by the ethics committee of the Ludwig-Maximilians-University, Munich, under the project-ID 338–15.

#### Neuroimaging

In OPTIMA we offer a basic neuroimaging protocol acquired on 3-Tesla clinical MRI system (General Electric, Milwaukee, USA) in order to extract information on macroscopic and microscopic brain features as well as brain function.

Neuroimaging in the context psychotherapy research regarding MDD is built on evidence that the clinically heterogeneous condition of MDD is reflected in structural and functional abnormalities of brain circuits [168, 169]. These abnormalities, on one hand, represent target systems that are modified by the learning processes stimulated by psychotherapy. On the other hand, heterogeneity of these abnormalities across subjects is expected and hypothesized to hold a predictive value with regard to the most effective type of psychotherapy for an individual [170]. This latter hypothesis will mainly be tested using a *response-status*-by-*treatment-type* interaction framework applied to extracted MRI features or to voxelwise/vertexwise measures. Analyses are designed *anatomically explorative* and will thus be controlled for multiple testing. Post-hoc analyses will comprise pair-wise group comparisons (e. g. responders of ST against non-responders of ST), comparisons of responders of one treatment against pooled non-responders, and a general responder/non-responder comparison. Examples for established feature extraction techniques are listed per MRI subdomain in the following:

1. A high resolution T1-weighted imaging with high contrast between grey matter, white matter and cerebrospinal fluid serves as basis for voxel-based and surface-based morphometry analyses using established imaging post-processing approaches. Discrete cortical thickness and surface area features, voxelwise volume maps and surface meshes will be calculated, and the above defined group comparisons performed for the entire anatomical space (either covered by extracted anatomical features or by voxels/vertex points).
2. Diffusion tensor imaging (DTI) is acquired in order to allow for the reconstruction of fiber tracks as basis for structural connectivity and to calculate voxel wise maps of measure of fiber integrity such as fractional anisotropy. DTI is suited to probe specific hypotheses on ‘hard-wired’ connectivity patterns of specific networks as anatomical basis of functional re-organization. Probabilistic region-by-region-connectivity values using the FreeSurfer cortical/subcortical parcellation for ROI-definition will represent the main target features of this domain.
3. Resting state functional MRI (rs-fMRI) over 6.5 min is acquired in an eyes-open-crosshair-fixation design with parallel eye-tracking. Respiration and pulse measurements are taken for later denoising steps. Resting state fMRI allows for different types of functional connectivity analyses at the whole brain level (e. g. functional connectivity density maps forwarded to second level analyses) or at the level of specific circuitries. For the latter, group independent component analysis will be used to extract a set of within-network and between-network connectivity using validated analysis pipelines.
4. Task fMRI: In order to acquire information on social interaction information processing, a shortened version of an established *social interaction task* is performed, which involves gaze contact with an interaction partner who reacts in real-time in the context of an object selection task (same image geometry and parameters as rs-fMRI for optimal coupling) [171, 172]. This task validly recruits specific neural systems that are involved in social processes and that are highly relevant to participation in psychotherapy. The above-mentioned group comparisons represent (voxelwise) second level analyses based on first level activation maps that hold information on the individual’s strength of the social network recruitment.

MRI measurements (1)–(3) will be repeated at post treatment, yet we expect dropouts here and thus refrained from building the main hypotheses on longitudinal MRI data. For details of the tested domains and applied procedures see Additional file 1: Appendix B.

#### Bayesian social learning task

Different psychotherapy approaches rely on basic processes of learning. This is particularly relevant for ST, which aims to overcome EMS, that is enable individuals to make new interpersonal experiences, and focuses on the therapeutic alliance and patient-therapist interaction [15, 27]. Therefore, we included a social learning task, that enables additional insights into the underlying learning and decision-making mechanisms, in other words, why and how participants learn and behave a certain way [173].

We use a reward-based learning task that requires the integration of non-social and social cues in conjunction with computational modeling [174]. In this task, participants have to learn about the winning probabilities of two cards in order to win points, which will be turned into a financial gain at the end of the study. In addition, a face in the center of the screen looks at one of the two cards, before the participant can make his/her choice. The probability of this gaze shift being helpful or not is also systematically manipulated. Both card and gaze probabilities fluctuate according to a fixed schedule, which is unbeknown to the participant, and do so independently from one another. Behavioral responses to this task are collected to assess participants’ performance in terms of total points achieved. Here, the impact of the non-social and the social domain can also be studied. Furthermore, computational modelling allows to assess learning and decision-making parameters estimated for each participant from their behavior. These parameters could help to shed new light onto psychotherapeutic processes, which also rely on social learning [175], and potentially help to predict treatment outcomes [176].

#### Sociodemographic, clinical and personality parameters

In the past, sociodemographic and clinical parameters have been used to predict outcome of the treatment of depression [177–179], but little is known about their associations and interplay with potential predictors as described above. Therefore, we include sociodemographic (such as age, gender, socioeconomic status, etc.), clinical parameters (such as comorbidities, symptom severity, etc.) and personality measures (Assessment of the DSM-IV Personality Disorders (ADP-IV) [180], the DSM-V Level of Personality Functioning Scale – Self Report (LPFS-SR) [181], the Personality Inventory for DSM-5 (PID-5) Short Version [182–184]), stressful and traumatic life events (Childhood Trauma Questionnaire (CTQ) [185], Social Readjustment Scale (SRRS) [186]), and motivation related constructs (Behavioral Inhibition System/Behavioral Activation System Scales (BIS/BAS) [187]). Data analysis and data management.

### Power and statistical analysis

Power estimation for the current investigation is based on the main research question on the effectiveness of ST and hypothesis H1 on the superiority of ST over IST regarding the primary outcome (BDI-II). We presume that the minimal clinically important difference (MCID) regarding BDI-II scores should be related to initial depression severity and a patient perspective of perceived improvement [188]. Button and colleagues estimated a minimum reduction of 17.5% of BDI-II scores as MCID. Based on our baseline pilot data, an effect size of d = 0.4 would allow us to detect all outcome differences that can be considered as MCID in our target sample which consists of moderate to severe depressed patients in an inpatient and day clinic setting. If setting power to 0.80 and *α* to 0.05 while using two-sided t-tests and following a 1:1:1 randomization to ST, CBT, or IST, it is necessary to recruit *n* = 99.1 per group resulting in an overall sample size of *N* = 300 (rounded) participants to identify differences in BDI of d = 0.4.

Regarding H2 on the non-inferiority of ST compared with CBT, a sample size of *n* = 100 per group, a one-sided significance level of α = 0.05, and setting power to 0.80 will allow us to evaluate a non-inferiority margin of d = .36. This is even lower than what can be considered as MCID [188] and takes into account the potential role of concurrent medication during the psychotherapy treatment.

We will apply different methods such as Holm procedure [189] in a scientifically appropriate manner regarding all future data analyses derived from the current study in order to prevent family wise error rate. The Holm method can be applied in same cases like Bonferroni correction to control for multiple testing, but is a more advanced and powerful tool [190].

For the analysis of primary and secondary outcome variables, we will apply linear mixed-effect models (e.g. to explore predictors) which outperform other approaches like analysis of covariance when data is missing not completely at random [191] . Additionally, linear mixed-effect models have increased power compared to simple linear regression approaches as the intra-class coefficient increases [192], thus, a targeted sample size of *N* = 300 is likely a conservative estimate to assess treatment effects with a power of 80% and an alpha level of 0.05. When investigating treatment outcome in clinical trials, the role of missing values needs to be considered. In order to deal with them adequately, we will follow an intention-to-treat approach using multiple imputation techniques [193] and use more specialized statistical analyses such as survival analyses e.g., to investigate dropout as a clinically relevant secondary outcome. For investigating specific and non-specific MOCs in ST, CBT, and IST treatment arm (H3 – H5), we will fit growth models within multilevel and structural equation model frameworks taking into account the nested structure of the data and potential meaningful growth over time [194, 195]. Multilevel models are able to differentiate within- from between-person variation considering the hierarchies within the data (such as time point of measurements within individuals) and by including random slopes and random intercepts [194, 196].

### Data management and monitoring

Data collection and management is conducted according to German law. Here, patient data is stored on encrypted institute servers in pseudonymized manner to restrict access to full details (i.e., personal identifying and study data) to dedicated study personnel only. In order to ensure data quality, double data entry is applied. If requested by a participant, all individual data is removed from all servers immediately. Data presented in publications will be fully anonymous and will not allow identification of study participants. Study documents will be kept at the Max Planck Institute of Psychiatry for the duration of the study and consecutive data analysis. All data that is not kept in the biobank, will be deleted 25 years after end of the study.

The occurrence of adverse events, defined as the development of acute suicidality, and serious adverse events, defined as suicide attempt, will lead to the immediate exclusion of the participant from any study procedures. In such cases, necessary psychiatric care will be provided. Serious adverse events are reported to the Institutional Ethic Committee of the Faculty of Medicine of Ludwig-Maximilians-University Munich. If they are related to study procedures, the study is terminated immediately.

Since it is a psychotherapy trial, blinding of participants and personnel (except from raters) in the OPTIMA trial is impossible and potential adverse events or deteriorations directly assignable to treatment conditions. Therefore, the establishement of a data monitoring committee, which is normally installed in masked trials to supervise adverse events and potential relations to the experimental treatment condition, is not necessary.

## Discussion

The acute and sustainable treatment of MDD is one of the most urgent health related challenges of our times, as it constitutes one of the leading causes of disability and disease burden today and in the future [197]. Comorbidities on axis I and II are associated with mood disorders [198] and play a key role as risk factor for recurrence of MDD [199]. Originally developed for non-responders of cognitive therapy and effective in the treating of PD [15, 33], ST represents a promising approach in the treatment of psychiatric disorders in general and MDD in particular. It focuses on the modification of trait-like EMS which play a key role in the development and maintenance of psychopathology. Therefore, ST is tested in the current trial in an inpatient and day clinic setting as a psychotherapy approach for MDD in its rather severe manifestations characterized by recurrence, comorbidities especially on axis II, and chronical courses.

The OPTIMA-Trial overcomes several short-comings of prior research projects on the effectiveness of ST. The design of the study combines external validity of a naturalistic inpatient and day clinic setting with methodological standards of clinical trials such as randomization procedure, allocation concealment, blinding of outcome assessments, adherence ratings and by conducting intention-to-treat analysis. Additionally, the study implements recommendations for psychotherapy RCTs [103] e.g., by applying treatments to a rather heterogeneous sample (e.g., with respect to age range and comorbidities), using an active control arm (IST), adding behavioral and biological markers, combining self-assessments and clinical assessments (M-CIDI or MADRS) as outcome variables, and following standardized/manualized psychotherapy protocols. Beyond this, we added two follow-up visits after study completion (at month 6 and 24) to gain insight into long-term effects of our interventions that are both clinically and economically relevant.

Nevertheless, this study faces some challenges and has methodological limitations. First, it is in the nature of psychotherapy trials, that the blinding of participants and personnel (except from raters) is impossible to achieve. Thus, therapy expectancy effects constitute a potential risk of bias [200]. Second, the study duration of 7 weeks could be judged as too short to capture lasting treatment effects of psychotherapy. Specifically, one might expect a greater impact of psychotherapy when it is administered for a longer period of time. Yet, considering the inpatient and day clinic sample of the trial, 7 weeks displays a realistic picture of the clinical reality rather than a longer stay at the hospital. To account for the short treatment phase, we plan two later assessments at 6 and 24 months post-treatment to cover these long-term effects. Third, from a pure methodological perspective, a “psychopharmacotherapy only” and/or a “psychopharmacotherapy free” treatment arm would enable us to detect the relative contributions of ST and ADM to the recovery more accurately. However, the combination of ADM and psychotherapy is considered as gold standard in the treatment guidelines of MDD [93]. Given that the participants in our sample suffer from rather severe forms of MDD at the time point when they are being enrolled in the study, we decided not to include a “psychotherapy only” or “psychopharmacotherapy only” arm in the study design for ethical reasons. Fourth, the semi-open structure of the group sessions means varying patient constellations and potential therapist change, which might disrupt therapy processes and the development of therapy alliance and group cohesion. Fifth, the measures in the current research are time consuming and demanding. Loss of motivation is a core feature of depression and therefore, the study procedures might bias the selection of participants and facilitate drop outs. Even though, we cannot totally rule out this limitation, we hope to lower such biases by reducing the obligatory measurements to a minimum and adapting measurement procedures to the participants’ schedule to make participation as convenient as possible. Beyond that, we will use CONSORT guidelines [201] to ensure a transparent reporting of the trial and detect and evaluate potential remaining sampling biases when interpreting results in terms of generalizability. Since the major parts of the measurements happen before and after treatment, we do not consider them to interfere with the process of psychotherapy. Sixth, psychotherapy is one element of comprehensive inpatient and day clinic treatment approach including ADM and further elements of psychiatric care. Nevertheless, as outlined above, we assume psychotherapy and specific intervention techniques like in ST to play a major role in the treatment of depression and therefore expect an additional effect to non-specific therapy (IST). In accordance with treatment guidelines [101] and to assure psychotherapy not to be neglected in the overall care, we choose a relatively high treatment dose when comparing ST with CBT. In order to avoid biases in favor a non-inferiority hypothesis, we chose a rather small non-inferiority margin and will take this aspect into account when interpreting the results. Furthermore, all additional therapies and ADM will be documented and considered for later use in the statistical analysis as potential confounder. Even though we assume the further elements of psychiatric care to average out between arms, improvement in symptom severity cannot be directly associated to the specific psychotherapy interventions alone. The inclusion of psychotherapy in daily psychiatric care constitutes a methodological challenge, but simultaneously provides the opportunity to test treatment effects in a realistic scenario. Finally, pilot data suggests that the application of the mentioned inclusion and exclusion criteria will result in a sample consisting of partly moderate, but mainly severe forms of MDD. Such a sample represents reality in an inpatient and day clinical settings, but has to be taken into account as a factor when generalizing the results.

In conclusion, by its multimodal character, the broad inclusion criteria, and randomization into standardized psychotherapy treatment arms, the OPTIMA Trial addresses key questions on how psychotherapy in treatment of MDD can be optimized in a realistic clinical setting and helps to gain insights into a better understanding of predictors and mechanisms of different approaches of psychotherapy.

## Trial status

This is the first version of the protocol. First patient was randomized in September 2017. The recruitment phase of the trial will be completed by end of 2022 including follow-up measurements.

## Supplementary information

**Additional file 1.**

## Data Availability

Not applicable.

## Abbreviations

MDD: Major Depressive Disorder
ADM: Antidepressant Medication
ST: Schema Therapy
CBT: Cognitive Behavioral Therapy
PD: Personality Disorder(s)
EMS: Early Maladaptive Schemas
IST: Individual Supportive Therapy
MOCs: Mechanisms of Change
ECG: Electrocardiography
EMA: Ecological Momentary Assessment
BDI-II: Beck Depression Inventory-II
MADRS: Montgomery-Asberg Depression Ratings Scale
M-CIDI: Munich-Composite International Diagnostic Interview
BSI: Brief Symptom Inventory
WHODAS: World Health Organization Disability Assessment Schedule
WHOQOL: World Health Organization Quality of Life
HR: Heart Rate
RNT: Repetitive Negative Thinking
HRV: Heart Rate Variability
DNA: Deoxyribonucleic Acid
RNA: Ribonucleic Acid
EDTA: Ethylenediamine Tetra acetic Acid
PBMCs: Peripheral Blood Mononuclear Cells
DMSO: Dimethyl Sulfoxide
fMRI: Functional Magnetic Resonance Imaging
DTI: Diffusion Tensor Imaging
MCID: Minimal Clinically Important Difference
