# Supplementary material for: Schema therapy versus cognitive behavioral therapy versus individual supportive therapy for depression in an inpatient and day clinic setting: study protocol of the OPTIMA-RCT
Source: BMC Psychiatry. 2020 Oct 14;20:506. doi: 10.1186/s12888-020-02880-x (PMC7557007; doi:10.1186/s12888-020-02880-x)
Supplement: Supplementary file 1 — Additional file 1. [file 12888_2020_2880_MOESM1_ESM.docx]

# Appendix A

## Psychometry

In addition to the already mentioned primary and secondary outcomes, the following questionnaires are used: Regarding MOCs and ST related process measures Young Schema Questionnaire – Short Version 3 (YSQ-S3) [1], Young Positive Schema Questionnaire (YPSQ) [2], Schema Mode Inventory (SMI) [3], the Working Alliance Inventory (WAI) [4], the Relationship Scales Questionnaire (RSQ) [5] and emotion regulation by the Emotion Regulation Questionnaire (ERQ) [6], affect by the Need for Affect Questionnaire (NAQ) [7] and the Positive and Negative Affect Schedule (PANAS) [8, 9] are assessed.

Cognition related aspects of depression are measured by the Automatic Thought Questionnaire (ATQ) [10], the Dysfunctional Attitude Scale (DAS) [11], the Internal and External Control Beliefs Scale (I-E-4) [12], the Cognitive Style Questionnaire – Short Form in German (CSQ-SF-D) [13] and self-efficacy by the *Allgemeine Selbstwirksamkeitsskala* (ASKU) [14].

Regarding treatment related questions and common factors we assess goal attainment by the Goal Attainment Scale (GAS) [15], therapy expectations by the Patient Questionnaire on Therapy Expectation and Evaluation (PATHEV) [16], mechanisms of change in psychotherapy by the Scale for the Multiperspective Assessment of General Change Mechanisms in Psychotherapy (SACiP) [17], and evaluate single sessions by the Session Evaluation Questionnaire (SEQ-D) [18]. PATHEV, SACiP, and WAI is used in a patient and therapist version and wording is minimally adapted, if necessary. SACiP is additionally adapted for group settings.

Additionally, we determine perceived stress by the Perceived Stress Scale (PSS) [19, 20], thought-action fusion in the context of suicidality (TAF) [21]. resilience with the Brief Resilience Scale (BRS) [22], and coping with depression by the Response Style Questionnaire (RSS/RSQ-D) [23].

Personality dispositions are evaluated through the Assessment of the DSM-IV Personality Disorders (ADP-IV) [24], the DSM-V Level of Personality Functioning Scale – Self Report (LPFS-SR) [25], the Personality Inventory for DSM-5 (PID-5) Short Version [26–28] and Behavioral Inhibition System/Behavioral Activation System Scales (BIS/BAS) [29]. Stressful and traumatic events are measured by the Childhood Trauma Questionnaire (CTQ) [30] and the Social Readjustment Scale (SRRS) [31]. We furthermore collect information on sleep habits with the Munich Chronotype Questionnaire (MCTQ) [32]. For details for time point of measurement see Table A1.

| **Table A1.** Overview on grouped psychometric measures. | | | | | | | | | | | | | |
| --- | --- | --- | --- | --- | --- | --- | --- | --- | --- | --- | --- | --- | --- |
|  |  | T0 | T1 | T2 | T3 | T4 | T5 | T6 | T7 | | T8 | | T9 |
| **Primary outcome for treatment comparison** | | | | | | | | | | | | | |
|  | BDI-II | X | X | X | X | X | X | X | X | | X | | X |
| **Secondary outcome for treatment comparison** | | | | | | | | | | | | | |
|  | MADRS | X |  |  |  | X |  |  | X | | X | | X |
|  | M-CIDI | X |  |  |  |  |  |  | X | | X | | X |
|  | WHODAS | X |  |  |  | X |  |  | X | | X | | X |
|  | WHOQOL | X |  |  |  |  |  |  | X | | X | | X |
|  | BSI | X | X | X | X | X | X | X | X | | X | | X |
| **MOCs and process measures** | | | | | | | | | | | | | |
|  | YSQ-S3 | X |  |  |  | X |  |  | X | | X | | X |
|  | YPSQ | X |  |  |  | X |  |  | X | | X | | X |
|  | SMI | X |  |  |  | X |  |  | X | | X | | X |
|  | WAI(P)^a^ |  |  |  |  | X^b^ |  |  | X^c^ | |  | |  |
|  | WAI(T)^b^ |  |  |  |  | X^b^ |  |  | X^c^ | |  | |  |
|  | RSQ | X |  |  |  | X |  |  | X | | X | | X |
|  | ERQ | X | X | X | X | X | X | X | X | | X | | X |
|  | NAQ | X | X | X | X | X | X | X | X | | X | | X |
|  | PANAS | X | X | X | X | X | X | X | X | | X | |  |
|  | ATQ | X | X | X | X | X | X | X | X | | X | | X |
|  | DAS | X | X | X | X | X | X | X | X | | X | | X |
|  | IE-4 | X | X | X | X | X | X | X | X | | X | | X |
|  | CSQ-SF-D | X | X | X | X | X | X | X | X | | X | | X |
|  | ASKU | X | X | X | X | X | X | X | X | | X | | X |
|  | GAS(P)^a^ |  | X |  |  | X |  |  | X | |  | |  |
|  | GAS(T)^b^ |  | X |  |  | X |  |  | X | |  | |  |
|  | PATHEV(P)^a^ |  | X^c^ |  |  | X^d^ |  |  |  | |  | |  |
|  | PATHEV(T)^b^ |  | X^c^ |  |  | X^d^ |  |  |  | |  | |  |
|  | SACIP(P)^a^ |  | X^c^ |  |  | X^d^ |  |  | X^e^ | |  | |  |
|  | SACIP(T)^b^ |  | X^c^ |  |  | X^d^ |  |  | X^e^ | |  | |  |
|  | SEQ-d |  | X^c^ |  |  | X^d^ |  |  | X^e^ | |  | |  |
| **Further MDD related measures** | | | | | | | | | | | | |  |
|  | PSS-4 | X | X | X | X | X | X | X | X | | X | | X |
|  | TAF-SR | X |  |  |  | X |  |  | X | |  |  |  |
|  | BRS | X |  | X |  | X |  |  | X | | X | | X |
|  | RSS/RSQ-D | X | X | X | X | X | X | X | X | | X | | X |
| **Potential predictors** | | | | | | | | | | | | | |
|  | ADP4 | X |  |  |  |  |  |  |  | | X | | X |
|  | PID-5-SE | X |  |  |  |  |  |  | X | | X | | X |
|  | SEFP-SR | X |  |  |  |  |  |  | X | |  |  |  |
|  | BIA/BAS | X |  |  |  |  |  |  | X | | X | X |  |
|  | CTQ | X |  |  |  |  |  |  |  | |  |  |  |
|  | SRRS | X |  |  |  |  |  |  |  | |  |  |  |
| **Sleep habits** | | | | | | | | | | | | | |
|  | MCTQ | X |  |  |  |  |  |  |  |  |  | |  |

*Note*: Time point T0 refers to study week 0 (pre-treatment), time point T1 refers to study week 1, etc. Time point T8 refers to follow up assessment after 6 months, T9 to follow up assessment after 2 years. ^a)^ Patient version. ^b)^ Therapist version. ^c)^ Information collected after 1^st^ session. ^d)^ Information collected after 7^th^ session. ^e)^ Information collected after 13^th^ session.

# Appendix C

| **Table C1.** Sequence details of MRI protocol (3 Tesla clinical MR750 scanner, General Electric) | |
| --- | --- |
| **Acquisition category** | **Sequence details** |
| (1) High resolution T1-weighted imaging | Sagittal FSPGR 3D BRAVO, TE 2.3 ms, TR 6.2 ms, TI 450 ms, FA 12°, FOV 25.6 x 25.6 x 20.0 cm^3^, matrix 256 × 256 x 200, FDir S/I |
| (2) Diffusion tensor imaging | 2D spin echo DTI, TE 60.9 ms, TR 8 s, interleaved/bottom-up, 66 diffusion directions, 5 initial B0 images, FOV 25.6 x 25.6 cm^2^, ST 2.0 mm, SP 0 mm, 60 slices, matrix 128 × 128, Fat Saturation |
| (3) Resting state functional MRI | 2D Gradient Echo EPI, oblique, AC-PC alignment, FA 90°, TE 30.0 ms, TR 2.5 s, interleaved/ bottom-up, no dummy scans, FOV 24.0 × 24.0 cm^2^, ST 3.0 mm, SP 0.5 mm, 42 slices, matrix 96 × 96, FDir R/L, acceleration factor 2, 155 volumes |
| (4) Social interaction task | Sequence parameters as (3), duration 15 minutes |
| *Note:* Adjunct B0 images using an inverted phase sampling are recorded for geometric unwarping steps. | |
|  |  |

References

1. Kriston L, Schäfer J, Jacob GA, Härter M, Hölzel LP. Reliability and Validity of the German Version of the Young Schema Questionnaire – Short Form 3 (YSQ-S3). European Journal of Psychological Assessment. 2013;29:205–12. doi:10.1027/1015-5759/a000143.

2. Louis JP, Wood AM, Lockwood G, Ho M-HR, Ferguson E. Positive clinical psychology and Schema Therapy (ST): The development of the Young Positive Schema Questionnaire (YPSQ) to complement the Young Schema Questionnaire 3 Short Form (YSQ-S3). Psychol Assess. 2018;30:1199–213. doi:10.1037/pas0000567.

3. Reiss N, Dominiak P, Harris D, Knörnschild C, Schouten E, Jacob GA. Reliability and Validity of the German Version of the Schema Mode Inventory. European Journal of Psychological Assessment. 2012;28:297–304. doi:10.1027/1015-5759/a000110.

4. Horvath AO, Greenberg LS. Development and validation of the Working Alliance Inventory. Journal of Counseling Psychology. 1989;36:223–33. doi:10.1037/0022-0167.36.2.223.

5. Steffanowski A, Oppl M, Meyerberg J, Schmidt J, Wittmann WW, Nübling R. Psychometrische Überprüfung einer deutschsprachigen Version des Relationship Scales Questionnaire (RSQ). In: Störungsspezifische Therapieansätze - Konzepte und Ergebnisse. p. 320–342.

6. Abler B, Kessler H. Emotion Regulation Questionnaire – Eine deutschsprachige Fassung des ERQ von Gross und John. Diagnostica. 2009;55:144–52. doi:10.1026/0012-1924.55.3.144.

7. Appel M, Gnambs T, Maio GR. A short measure of the need for affect. J Pers Assess. 2012;94:418–26. doi:10.1080/00223891.2012.666921.

8. Krohne HW, Egloff B, Kohlmann C-W, Tausch A. Untersuchungen mit einer deutschen Version der "Positive and Negative Affect Schedule" (PANAS) 1996.

9. Watson D, Clark LA, Tellegen A. Development and validation of brief measures of positive and negative affect: The PANAS scales. Journal of Personality and Social Psychology. 1988;54:1063–70. doi:10.1037/0022-3514.54.6.1063.

10. Hollon SD, Kendall PC. Cognitive self-statements in depression: Development of an automatic thoughts questionnaire. Cogn Ther Res. 1980;4:383–95. doi:10.1007/BF01178214.

11. Hautzinger M, Joormann J, Keller F. DAS - Skala dysfunktionaler Einstellungen. Göttingen: Hogrefe Verlag; 2005.

12. Kovaleva A, Beierlein, C., Kemper,C.J., Rammstedt B. Eine Kurzskala zur Messung von Kontrollüberzeugungen: Die Skala Internale-Externale-Kontrollüberzeugung-4 (IE-4). GESIS-Working Papers. 2012.

13. Huys QJM, Renz D, Petzschner F, Berwian I, Stoppel C, Haker H. German Translation and Validation of the Cognitive Style Questionnaire Short Form (CSQ-SF-D). PLoS ONE. 2016;11:e0149530. doi:10.1371/journal.pone.0149530.

14. Beierlein, C., Kovaleva, A., Kemper, C.J. & Rammstedt, B. (2012). Allgemeine Selbstwirksamkeit Kurzskala ASKU - Manual: Gesis.

15. Kiresuk TJ, Sherman RE. Goal attainment scaling: A general method for evaluating comprehensive community mental health programs. Community Ment Health J. 1968;4:443–53. doi:10.1007/BF01530764.

16. Schulte D. Messung der Therapieerwartung und Therapieevaluation von Patienten (PATHEV). Zeitschrift für Klinische Psychologie und Psychotherapie. 2005;34:176–87. doi:10.1026/1616-3443.34.3.176.

17. Mander JV, Wittorf A, Schlarb A, Hautzinger M, Zipfel S, Sammet I. Change mechanisms in psychotherapy: Multiperspective assessment and relation to outcome. Psychother Res. 2013;23:105–16. doi:10.1080/10503307.2012.744111.

18. Hartmann A, Leonhart R, Hermann S, Joos A, Stiles WB, Almut Zeeck. Die Evaluation von Therapiesitzungen durch Patienten und Therapeuten. Diagnostica. 2013;59:45–59. doi:10.1026/0012-1924/a000078.

19. Cohen S, Kamarck T, Mermelstein R. A global measure of perceived stress. Journal of Health and Social Behavior. 1983;24:385–96.

20. Klein EM, Brähler E, Dreier M, Reinecke L, Müller KW, Schmutzer G, et al. The German version of the Perceived Stress Scale - psychometric characteristics in a representative German community sample. BMC Psychiatry. 2016;16:159. doi:10.1186/s12888-016-0875-9.

21. Gjelsvik B, Kappelmann N, Soest T von, Hinze V, Baer R, Hawton K, Crane C. Thought-Action Fusion in Individuals with a History of Recurrent Depression and Suicidal Depression: Findings from a Community Sample. Cogn Ther Res. 2018;42:782–93. doi:10.1007/s10608-018-9924-7.

22. Chmitorz A, Wenzel M, Stieglitz R-D, Kunzler A, Bagusat C, Helmreich I, et al. Population-based validation of a German version of the Brief Resilience Scale. PLoS ONE. 2018;13:e0192761. doi:10.1371/journal.pone.0192761.

23. Kühner C, Huffziger S, Nolen-Hoeksema S. RSQ-D: Response Styles Questionnaire - Deutsche Version. Göttingen: hogrefe; 2007.

24. Doering S, Renn D, Höfer S, Rumpold G, Smrekar U, Janecke N, et al. Validierung der deutschen Version des Fragebogens zur Erfassung von DSM-IV Persönlichkeitsstorungen (ADP-IV). Z Psychosom Med Psychother. 2007;53:111–28. doi:10.13109/zptm.2007.53.2.111.

25. Morey LC. Development and initial evaluation of a self-report form of the DSM-5 Level of Personality Functioning Scale. Psychol Assess. 2017;29:1302–8. doi:10.1037/pas0000450.

26. Markon KE, Quilty LC, Bagby RM, Krueger RF. The development and psychometric properties of an informant-report form of the personality inventory for DSM-5 (PID-5). Assessment. 2013;20:370–83. doi:10.1177/1073191113486513.

27. Zimmermann J, Altenstein D, Krieger T, Holtforth MG, Pretsch J, Alexopoulos J, et al. The structure and correlates of self-reported DSM-5 maladaptive personality traits: findings from two German-speaking samples. J Pers Disord. 2014;28:518–40. doi:10.1521/pedi_2014_28_130.

28. Zimmermann J, Böhnke JR, Eschstruth R, Mathews A, Wenzel K, Leising D. The latent structure of personality functioning: Investigating criterion a from the alternative model for personality disorders in DSM-5. J Abnorm Psychol. 2015;124:532–48. doi:10.1037/abn0000059.

29. Strobel A, Beauducel A, Debener S, Brocke B. Eine deutschsprachige Version des BIS/BAS-Fragebogens von Carver und White. Zeitschrift für Differentielle und Diagnostische Psychologie. 2001;22:216–27. doi:10.1024//0170-1789.22.3.216.

30. Bernstein DP, Fink L. Childhood Trauma Questionnaire: A retrospective self-report manual. San Antonio: TX: The Psychological Corporation; 1998.

31. Holmes TH, Rahe RH. The social readjustment rating scale. Journal of Psychosomatic Research. 1967;11:213–8. doi:10.1016/0022-3999(67)90010-4.

32. Roenneberg T, Keller LK, Fischer D, Matera JL, Vetter C, Winnebeck EC. Human activity and rest in situ. Meth Enzymol. 2015;552:257–83. doi:10.1016/bs.mie.2014.11.028.
